# Hand hygiene practices during the COVID-19 pandemic and associated factors among barbers and beauty salon workers in Ethiopia

**DOI:** 10.1371/journal.pone.0269225

**Published:** 2022-07-01

**Authors:** Tarikuwa Natnael, Metadel Adane, Solomon Goraw

**Affiliations:** 1 Department of Environmental Health, College of Medicine and Health Sciences, Wollo University, Dessie, Ethiopia; 2 School of Law, Wollo University, Dessie, Ethiopia; National Health Laboratory Service, SOUTH AFRICA

## Abstract

Coronavirus disease-2019 (COVID-19) is still causing morbidity and mortality all over the world. Preventive measures such as wearing a facemask, social distancing and hand hygiene continue to be the only options available in countries such as Ethiopia where vaccines are not yet widely available. Hand hygiene is one of the easiest and cheapest preventive measures, and one that is especially important for barbers and beauty salon workers who are widely exposed to the virus due to their contact with many customers. Therefore, measuring the proportion of good hand hygiene practices and associated factors among barbers and beauty salon workers may provide essential guidance in the development of effective interventions to improve COVID-19 prevention measures. A facility-based cross-sectional study was conducted among 410 barbers and beauty salon workers in Dessie City and Kombolcha Town from January 5 to February 10, 2021. The study participants were selected using a simple random sampling technique. A structured questionnaire and an observational checklist were used to collect the data. The collected data were entered into EpiData version 4.6 and analysed using Statistical Package for Social Sciences (SPSS) version 25.0. Logistic regression analysis using bivariate and multivariable logistic regression models was employed. From the bivariate analysis, variables with *p* <0.25 were retained into multivariable logistic regression analysis. Finally, from the multivariable analysis, variables that had a *p*-value < 0.05 were declared as factors significantly associated with good hand hygiene practices. Of the total 410 barbers and beauty salon workers, 52.9% [95% CI: 48.3–57.6] had good hand hygiene practices whereas 47.1% [95% CI: 42.4–51.7] had poor hand hygiene practices. From the total respondents, more than half 250 (61%) were male and 160 (39%) were female, with a mean age of 27.42 ±7.37 years. Out of 410 barbers and beauty salon workers, 73.7% had good knowledge about COVID-19 and 59.5% had a positive attitude towards taking precautions against COVID-19. Female sex (AOR = 2.17, 95% CI:1.29–3.65), educational level of college or above (AOR = 5.53, 95% CI:2.85–10.71), positive attitude towards taking precautions against COVID-19 (AOR = 2.4, 95% CI:1.46–4.17), belief in the effectiveness of hand hygiene practices (AOR = 3.78, 95% CI:2.18–6.55) and presence of a hand-washing facility with soap and water (AOR = 5.55, 95% CI:3.28–9.40) were factors significantly associated with good hand hygiene practices among barbers and beauty salon workers. The proportion of good hand hygiene practice was not sufficient to combat the virus. Good hand hygiene practice was higher among those with higher educational level, positive attitude towards taking precautions against COVID-19, belief in the effectiveness of hand hygiene practices, presence of a hand-washing facility with soap and water and those of female sex. Thus, improving hand hygiene practices through continued training, especially for those with a lower educational level and for male workers, is recommended. Moreover, government and non-government organizations should work together to provide alcohol-based hand sanitizer at a low cost to those barbershops and beauty salons if there is no access to water and soap.

## Introduction

In December 2019, coronavirus infectious disease-19 (COVID-19), caused by the severe acute respiratory syndrome coronavirus-2 (SARS-CoV-2) was identified in Wuhan, China [1]. Since then, the virus has spread rapidly around the world, with a huge impact on human health and the world economy. Many countries have struggled to apply various strategies against the pandemic, even while more than 108.2 million infections and over 2.3 million deaths were reported globally as of 14^th^ February 2021 [2]. It was estimated that due to COVID-19, gross domestic product (GDP) would fall by 2% overall around the world in 2020, 2.5% in developing countries and 1.8% in industrial countries [3].

COVID-19 is highly transmissible, including from asymptomatic individuals [4] through respiratory droplets and by touching a surface or object infected with the virus and then touching eyes, nose or mouth [5, 6]. Individuals with COVID-19 have shown symptoms such as fever, fatigue, dry cough, malaise and breathing difficulty [7–9]. The virus can also cause damage to tissues and organs of the infected host and lead to severe disease, including hospitalization, admission to an intensive care unit and death [10]. Severe cases and death have occurred mainly in older adults and people with chronic illnesses such as hypertension, cardiovascular disease, chronic kidney disease and diabetes [11–13]. Preventing these adverse impacts requires the application of measures such as hand hygiene, facemask wearing, cleaning and disinfecting frequently touched surfaces, staying home as much as possible and avoiding close contact with others [5].

Studies have also shown that non-pharmaceutical measures such as hand hygiene and facemasks are the easiest and most effective methods to reduce the transmission of respiratory infections [14, 15]. Hand hygiene has the potential to reduce the spread of respiratory infection by 16% [16]. It is also been shown that hand hygiene can reduce the transmission of gastrointestinal illness (GI) by 31.0% [17]. Washing hands frequently with water and soap for at least 20 seconds, and when water is not available, using alcohol-based hand sanitizers is crucial in the prevention of COVID-19 [5, 18].

Although hand hygiene is the cheapest and most effective method to prevent respiratory disease and GI illness, it was practised with higher compliance rates during the early phase of COVID-19 than more recently [19]. The rate of hand hygiene compliance was also observed to increase after the onset of the COVID-19 pandemic compared with that of pre-COVID-19 times.

A study in Germany showed increased adherence to hand hygiene practices from 47% before to 95% during the COVID-19 pandemic [20]. Similar observations were also made among Polish adolescents whose hand hygiene practices increased from 35.6% to 54.8% during the outbreak [21]. In Pakistan, furthermore, increased hand hygiene practices during the COVID-19 pandemic among healthcare workers has led to a reduction in hospital-associated infections (HAIs) in a hospital setting [22]. Even so, hand hygiene practices were higher during the early phase of the outbreak and then fell again among community members including barbers and beauty salon workers to that of pre-COVID-19 time.

Overall compliance with good hand hygiene practices is low, especially in developing countries where the reported compliance rates is 20.49% [23], while the risk of morbidity and mortality continued in the African region. Africa recorded 2,723, 431 COVID-19 positive cases and 68,294 deaths as of 14 February 2021 with the highest-burden in South Africa followed by Zambia and Nigeria [2].

Ethiopia is one of the developing countries with challenges in facing COVID-19 spread due to mass use of public transportation, a shortage of sanitation material, suspected cases being hidden, a lack of personal protective equipment for health care providers and the presence of immune-compromised people [24]. Although vaccines for the virus are available in some countries, in a resource-limited country such as Ethiopia it may take a long time to reach everyone, so applying preventive measures is the best and only option in tackling the virus.

The first confirmed case of COVID-19 in Ethiopia was registered in early March 2020 and then cases spread throughout the country. To tackle the problem, the government of Ethiopia advocated preventive measures such as avoiding handshakes, reducing the number of passengers riding public transportation by half, keeping adequate physical distancing, providing cleaning and hand washing facilities in every public institution [25]. While Ethiopia has responded to COVID-19 by taking various prevention measures, the virus has continued to cause morbidity and mortality, resulting in 145,704 cases and 2,181 deaths recorded as of 14 February 2021 [2].

Barbers and beauty salon workers are among those at the highest risk of getting COVID-19, but their level of hand hygiene practices is not well known. Thus, this study aimed to assess hand hygiene practices during the COVID-19 pandemic and associated factors among barbers and beauty salon workers in Dessie City and Kombolcha Town, Ethiopia. Understanding these factors may guide strategies related to increasing hand hygiene compliance and preventing the transmission of COVID-19 among barbers and beauty salon workers and the community.

## Materials and methods

### Study area

The study was conducted in Dessie City and Kombolcha Town in South Wollo Zone, one of the twelve zones found in Amhara Regional State. Dessie City is 401 km from Ethiopia’s capital City of Addis Ababa, and is at an elevation between 2,470 and 2,550 meters above sea level, while Kombolcha Town is 377 km away from Addis Ababa at an elevation of 1,857 meters above sea level. Based on the 2007 population and housing census projection, the total population of Dessie City was 212,436 and of Kombolcha Town of 126,144 [26].

### Study design, period and population

A facility-based cross-sectional study was conducted from January 5 to February 10, 2021 among barbers and beauty salon workers in Dessie City and Kombolcha Town. The source population was all barbers and beauty salon workers in Dessie City and Kombolcha Town. The study population was all systematically selected barbers and beauty salon workers who worked at the barbershops and beauty salons during the study period.

### Sample size determination and sampling technique

The single population proportion formula was used to determine the sample size [27]. The study used the assumptions of good hand hygiene practices among barbers and beauty salon workers at 50% since there is no previous study in a similar setting, Zα/2 value 1.96 at 95% confidence interval (CI) and 5% margin of error.


$$n=\frac{{\left(z_{a/2}\right)}^{2}*p(1-p)}{d^{2}}$$

The calculated sample size was 384; after considering a 10% non-response rate the final sample size became 422.

The total sample size was proportionally distributed between Dessie City and Kombolcha Town barbershops and beauty salons. Barbershops and beauty salons were then selected using a systematic sampling technique. One worker was selected randomly from each selected barbershop or beauty salon when there was more than one worker in a given location.

### Operational definitions

#### Hand hygiene practices

Compliance with the practices of cleansing hands with soap and water or with an antiseptic hand rub to remove transient microorganisms from hands [28].

#### Good hand hygiene practices

Study participants who correctly answered a number of questions greater than or equal to the mean from 11 total questions about hand hygiene practices using clean water and soap or alcohol-based hand sanitizer [29].

#### Poor hand hygiene practices

Study participants who correctly answered a number of questions fewer than the mean from 11 total questions about hand hygiene practices using clean water and soap or alcohol-based hand sanitizer [29].

#### Good knowledge

Study participants who correctly answered more than or equal to the mean number out of 14 total knowledge questions [30, 31].

#### Poor knowledge

Study participants who correctly answered fewer than the mean number out of 14 total knowledge questions [30, 31].

#### Positive attitude towards taking precautions against COVID-19

Study participants who scored higher than or equal to the mean out of 10 attitude questions [30, 31].

#### Negative attitude towards taking precautions against COVID-19

Study participants who scored lower than the mean on 10 attitude questions [30, 31].

### Data collection procedures and quality assurance

An interviewer-administered structured questionnaire and observational checklist were used to collect data. The questionnaire was adapted from previously published articles [21, 32, 33]. Socio-demographic and economic factors, knowledge about COVID-19, attitude towards taking precautions against COVID-19, behavioral and environmental factors were incorporated into the questionnaire. The questionnaire was originally prepared in English and then translated to the local language Amharic and back to English to ensure consistency.

Prior to the actual data collection, the questionnaire was pre-tested in a group of 5% of the total sample size in Kemisse Town. The result of the pre-test was used to correct some unclear ideas and statements. Four data collectors and two supervisors were involved in the study. One day of training was given to data collectors and supervisors. The data were collected by face-to-face interviews at the worksite and by observing the presence or absence of a hand-washing facility with soap and water and/or alcohol-based hand sanitizer. The questionnaires were checked daily for completeness and consistency by supervisors and the principal investigator. In addition, data entry errors were controlled through double data entry of a randomly selected 5% of the questionnaires.

### Data management and analysis

The collected data were coded and entered into EpiData version 4.6 and exported to SPSS version 25.0 for data cleaning and analysis. Descriptive statistics were calculated to describe the study populations using measures of frequency, percentages and proportions and were displayed using tables. The proportion of good hand hygiene practices among barbers and beauty salon workers was determined by dividing the number of workers with good hand hygiene practices by the total number of study participants.

Due to the binary nature of the outcome variable, binary logistic regression analysis was used. Variables that had a *p*-value < 0.25 by the bivariate analysis were then analysed by multivariable binary logistic regression to control the potential confounders. From the multivariable analysis, variables that had a *p*-value < 0.05 were declared as factors significantly associated with good hand hygiene practices. Model fitness was checked using Hosmer and Lemeshow goodness-of-fit-test, finding a *p*-value of 0.938.

### Ethics approval and consent to participate

Ethical clearance was obtained from the ethical review committee of Wollo University College of Medicine and Health Sciences with a protocol number CMHS/544/01/2021. Official permission letters were obtained from Dessie City and Kombolcha Town Health Bureaus. Prior to beginning the study, its purpose was explained to each participant and written consent was obtained from all participants. Participants were made aware that they had full right to participate or not in the study as well as to withdraw anytime during the interview. Confidentiality was also maintained through anonymity. During data collection, the data collectors wore facemasks, used alcohol-based hand sanitizer and kept a minimum of one meter distance from the interviewees to prevent transmission of the COVID-19 virus.

## Results

### Socio-demographic and economic characteristics

A total of 410 barbers and beauty salon workers were included in this study, from whom we yielded a response rate of 97.0%. More than half 250 (61%) of the barbers and beauty salon workers were male and 160 (39%) were female. Nearly two-thirds 263 (64.1%) of the barbers and beauty salon workers were aged 18–29 years and less than a tenth 36 (8.8%) were ≥40 years; the mean age was 27.42 years (±7.37SD). A primary-level education was reported by half 206 (50.2%) of the barbers and beauty salon workers while nearly one-fourth 94 (22.9%) had an education at college level or above (Table 1).

**Table 1:** Socio-demographic and economic characteristics of barbers and beauty salon workers in Dessie City and Kombolcha Town, Northeastern Ethiopia, January 5 to February 10, 2021.

Variables	Response	Frequency (*N = 410*)	Percentage (%)
**Sex**	Male	250	61
	Female	160	39
**Age (years)**	18–29	263	64.1
	30–39	111	27.1
	≥40	36	8.8
**Educational level**	Primary (up to grade 8)	169	41.2
	Secondary (grades 9–12)	123	30
	College or above	118	28.8
**Marital status**	Single	176	42.9
	Married	156	38
	Widowed	18	4.4
	Divorced	60	14.6
**Monthly income (USD)**	≤$152.60	260	63.4
	>$152.60	150	36.6
**Household size (persons)**	≤5	380	92.7
	>5	30	7.3
**Number of workers in the beauty salon/ barbershop**	≤2	287	70
	>2	123	30
**Number of customers per day**	≤11	305	74.4
	>11	105	25.6
**Work experience (years)**	1–2	69	16.8
	3–5	81	19.8
	>5	260	63.4
**Training about COVID-19**	Yes	47	11.5
	No	363	88.5
**Has chronic illness**	Yes	37	9
	No	373	91
**Availability of COVID-19 IPC guideline**	Yes	101	24.6
	No	309	75.4
**Knowledge about COVID-19**	Good	302	73.7
	Poor	108	26.3
**Attitude towards taking precautions against COVID-19**	Positive	244	59.5
	Negative	166	40.5
Mean barbers and beauty salon workers age (years) 27.42((±7.37SD)			

USD = United States Dollars, IPC = Infection Prevention and Control, SD = Standard deviation

### Knowledge and attitude status about COVID-19

Our findings showed that nearly three-fourths 73.7% [95% CI: 69.5–77.8] of the barbers and beauty salon workers had good knowledge about COVID-19, whereas just over one-fourth 26.3% [95% CI: 22.2–30.5] of them had poor knowledge. The response of the participants to questions about their attitude towards taking precautions against COVID-19 revealed that more than half 59.5% [95% CI: 55.1–64.1] of the workers had a positive attitude and nearly half 47.1% [95% CI: 42–52.2] of the workers had a negative attitude towards taking precautions against COVID-19.

### Behavioral and environmental factors

Of 410 barbers and beauty salon workers, nearly two-thirds 267 (65.1%) of the workers believed in the effectiveness of hand hygiene in preventing COVID-19 while just over one-third 143 (34.9%) of them did not believe in the effectiveness of hand hygiene. In this study, most 283 (69.5%) of the workers believed that there are no curative treatments for COVID-19. Just over half 211 (51.5%) of the workers perceived that they were vulnerable to COVID-19 and nearly half 199 (48.5%) did not feel vulnerable to the virus. With regard to environmental factors, in most 225 (54.9%) of the barbershops and beauty salons, there was a water source close by. In this study, nearly half 203 (49.5%) of the barbershops and beauty salons had a hand-washing facility with water and soap and the remaining 207 (50.5%) had no hand-washing facility (Table 2).

**Table 2:** Behavioral and environmental factors among barbers and beauty salon workers in Dessie City and Kombolcha Town, Northeastern Ethiopia, January 5 to February 10, 2021.

Variables	Response	Frequency (*N = 410*)	Percentage (%)
**Belief in the effectiveness of hand hygiene in preventing COVID-19**	Yes	267	65.1
	No	143	34.9
**Belief that there are no curative treatments for COVID-19**	Yes	283	69
	No	127	31
**Perceives self as vulnerable to COVID-19**	Yes	199	48.5
	No	211	51.5
**Worries about COVID-19**	Yes	224	54.6
	No	186	45.4
**Perceives that the consequence of getting COVID-19 is serious**	Yes	199	48.5
	No	211	51.5
**Experiencing any respiratory infection symptoms**	Yes	174	42.4
	No	236	57.6
**Has any acquaintances that experienced any respiratory infection symptoms**	Yes	180	43.9
	No	230	56.1
Knows someone who had positive test results for COVID-19	Yes	219	53.4
	No	191	46.6
Knows someone who was hospitalized for severe illness or died from COVID-19	Yes	216	52.7
	No	194	47.3
**Has young children in their household**	Yes	226	55.1
	No	184	44.9
**Family members encourage hand washing**	Yes	227	55.4
	No	183	44.6
**Healthcare workers encourage hand washing**	Yes	242	59
	No	168	41
**Presence of water source close to barbershop** and beauty salon	Yes	225	54.9
	No	185	45.1
**Experiencing a shortage of water**	Yes	224	54.6
	No	186	45.4
**Presence of improved latrine in/near the barbershop/beauty salon**	Yes	216	52.7
	No	194	47.3
**Presence of privately owned latrine in/near the barber shop/beauty salon**	Yes	209	51
	No	201	49
**Presence of hand-washing facility with water and soap**	Yes	203	49.5
	No	207	50.5
**Presence of poster showing hand-washing behaviour**	Yes	196	47.8
	No	214	52.2
**Presence of hand-washing facility that is convenient and user friendly in/near the barbershop/beauty salon**	Yes	204	49.8
	No	206	50.2

### Hand hygiene practices

The proportion of good hand hygiene practice among barbers and beauty salon workers was 52.9% [95% CI: 48.5–57.9]. One-fourth 104 (25.4%) of the barbers and beauty salon workers always practised hand hygiene before putting on a facemask and almost one-third 123 (30%) of them always did so after removing a facemask. Only 88 (21.5%) of the workers always practised good hand hygiene after coughing, sneezing, or blowing their noses (Table 3).

**Table 3:** Hand hygiene practices among barbers and beauty salon workers in Dessie City and Kombolcha Town, Northeastern Ethiopia, January 5 to February 10, 2021.

Variables	Response	Frequency (*N = 410*)	Percentage (%)
**Practice hand hygiene before putting on a facemask**	Always	104	25.4
	Sometimes	183	44.6
	Never	123	30
**Practice hand hygiene after removing a facemask**	Always	123	30
	Sometimes	198	48.3
	Never	89	21.7
**Practice hand hygiene after** coughing, sneezing, or blowing nose	Always	88	21.5
	Sometimes	144	35.1
	Never	178	43.4
**Practice hand hygiene after coming in contact with frequently touched surfaces/objects**	Always	86	21
	Sometimes	263	64.1
	Never	61	14.9
**Practice hand hygiene after coming in contact with coins/birr notes**	Always	96	23.4
	Sometimes	257	62.7
	Never	57	13.9
**Practice hand hygiene after using a latrine**	Always	158	38.5
	Sometimes	218	53.2
	Never	34	8.3
**Practice hand hygiene before eating**	Always	366	89.3
	Sometimes	30	7.3
	Never	14	3.4
**Practice hand hygiene after using public transportation**	Always	150	36.6
	Sometimes	209	51
	Never	51	12.4
**Practice hand hygiene after returning home**	Always	130	31.7
	Sometimes	237	57.8
	Never	43	10.5
**Practice hand hygiene before providing service to customers**	Always	135	32.9
	Sometimes	210	51.2
	Never	65	15.9
**Practice hand hygiene after providing service to customers**	Always	134	32.7
	Sometimes	222	54.1
	Never	54	13.2

Out of the total 410 barbers and beauty salon workers, only 80 (19.5%) practised hand hygiene by washing with water only. Nearly one-fourth 100 (24.4%) used water and soap and more than half 230 (56.1%) used, at various times, either water and soap or alcohol-based hand sanitizer. In the majority 254 (62%) of the barbershops and beauty salons, alcohol-based hand sanitizer was available; and more than two-thirds 290 (70.7%) of barbers/salon workers washed their hands for ≥20 seconds (Table 4).

**Table 4:** Hand hygiene practice-related factors among barbers and beauty salon workers in Dessie City and Kombolcha Town, Northeastern Ethiopia, January 5 to February 10, 2021.

Variables	Response	Frequency (*N = 410*)	Percentage (%)
**Methods of keeping hand hygiene**	Washing with water	80	19.5
	Washing with water and soap	100	24.4
	Washing with water and soap and/or using alcohol-based hand sanitizer	230	56.1
**Presence of alcohol-based hand sanitizer inside the barbershop/beauty salon**	Yes	254	62
	No	156	38
**Duration of hand hygiene procedure (seconds)**	<20	120	29.3
	≥20	290	70.7
**Frequency of washing hands (per day)**	1–2 times	30	7.3
	3–5 times	99	24.1
	6–10 times	155	37.8
	11–15 times	47	11.5
	16–20 times	40	9.8
	21–30 times	25	6.1
	>30 times	14	3.4
**Demonstrates practical hand-washing procedure perfectly**	Yes	225	54.9
	No	185	45.1

### Multivariable analysis of factors associated with good hand hygiene practices

From the bivariate analysis, sex, age, educational level, household size, work experience, presence of IPC guidelines, knowledge about COVID-19, attitude towards taking precautions against COVID-19 (Table 5), belief in the effectiveness of hand hygiene practices and presence of a hand-washing facility with soap and water were retained into multivariable analysis since these variables had a *p*-value < 0.25 from the bivariate analysis (Table 6).

**Table 5:** Factors associated with good hand hygiene practices among barbers and beauty salon workers in bivariate logistic regression analysis in Dessie City and Kombolcha Town, Northeastern Ethiopia, January 5 to February 10, 2021.

Variables	Response	Hand hygiene practices		COR (95%CI)	*P-*value
		Good	Poor		
		n	n		
**Sex**	Male	99	151	Ref	
	Female	118	42	4.28(2.77–6.61)	<0.001
**Age (years)**	18–29	133	130	Ref	
	30–39	59	52	1.10(0.71–1.72)	0.648
	≥40	25	11	2.22(1.05–4.69)	0.037
**Educational level**	Primary (up to grade 8)	80	89	Ref	
	Secondary (grades 9–12)	50	73	0.76(0.47–1.21)	0.257
	College or above	87	31	3.12(1.87–5.19)	<0.001
**Marital status**	Single	94	82	Ref	
	Married	78	78	0.87(0.56–1.34)	0.535
	Widowed	10	8	1.09(0.41–2.89)	0.862
	Divorced	35	25	1.22(0.67–2.20)	0.508
**Monthly income (USD)**	≤$152.60	140	120	Ref	
	>$152.60	77	73	0.90(0.60–1.35)	0.623
**Household size (persons)**	≤5	196	184	0.45(0.20–1.02)	0.057
	>5	21	9	Ref	
**Number of workers in the barbershop/ beauty salon**	≤2	152	136	0.99(0.65–1.52)	0.983
	>2	65	58	Ref	
**Number of customers per day**	≤11	163	142	1.08(0.69–1.69)	0.721
	>11	54	51	Ref	
**Work experience (years)**	1–2	28	41	Ref	
	3–5	41	40	1.50(0.78–2.87)	0.220
	>5	148	112	1.93(1.12–3.31)	0.016
**Presence of IPC guidelines**	Yes	60	41	1.41(0.89–2.23)	0.134
	N	157	152	Ref	
**Has chronic illness**	Yes	17	20	0.73(0.37–1.44)	0.735
	No	200	173	Ref	
**Training about COVID-19**	Yes	28	19	1.35(0.73–2.51)	0.333
	No	189	174	Ref	
**Knowledge about COVID-19**	Good	173	129	Ref	
	Poor	44	64	1.95(1.24–3.04)	0.003
**Attitude towards taking precautions against COVID-19**	Positive	159	85	3.48(2.30–5.26)	<0.001
	Negative	58	108	Ref	

Ref, reference category; COR, crude odds ratio; CI, confidence interval

**Table 6:** Factors associated with good hand hygiene practices among barbers and beauty salon workers in bivariate logistic regression analysis in Dessie City and Kombolcha Town, Northeastern Ethiopia, January 5 to February 10, 2021.

Variables	Response	Hand hygiene practices		COR (95%CI)	*P-*value
		Good	Poor		
		n	n		
**Belief in the effectiveness of hand hygiene in preventing COVID-19**	Yes	182	85	6.60(4.17–10.46)	<0.001
	No	35	108	Ref	
**Belief that there are no curative treatments for COVID-19**	Yes	153	130	1.15(0.76–1.76)	0.491
	No	64	63	Ref	
**Perceives self as vulnerable to COVID-19**	Yes	110	89	1.20(0.81–1.77)	0.355
	No	107	104	Ref	
**Worries about COVID-19**	Yes	118	106	0.97(0.66–1.44)	0.912
	No	99	87	Ref	
**Perceives that the consequence of getting COVID-19 is serious**	Yes	107	92	1.06(0.72–1.57)	0.740
	No	110	101	Ref	
**Experiencing any respiratory infection symptoms**	Yes	87	87	0.81(0.55–1.20)	0.308
	No	130	106	Ref	
**Has any acquaintances that experienced any respiratory infection symptoms**	Yes	92	88	0.87(0.59–1.29)	0.515
	No	125	105	Ref	
**Knows someone who had positive test results for COVID-19**	Yes	120	99	1.17(0.79–1.73)	0.417
	No	97	94	Ref	
**Knows someone who was hospitalized for severe illness or died from COVID-19**	Yes	116	100	1.06(0.72–1.57)	0.739
	No	101	93	Ref	
**Has young children in the household**	Yes	124	102	1.19(0.80–1.75)	0.383
	No	93	91	Ref	
**Family members encourage hand-washing**	Yes	125	102	1.21(0.82–1.79)	0.334
	No	92	91	Ref	
**Healthcare workers encourage hand-washing**	Yes	133	109	1.22(0.82–1.81)	0.323
	No	84	84	Ref	
**Presence of water source close to the household**	Yes	123	102	1.16(0.79–1.72)	0.436
	No	94	91	Ref	
**Experiencing a shortage of water**	Yes	123	101	Ref	
	No	94	92	1.19(0.80–1.76)	0.377
**Presence of improved latrine in/near the barbershop/beauty salon**	Yes	116	100	1.06(0.72–1.57)	0.739
	No	101	93	Ref	
**Presence of privately owned latrine in/near the barbershop/beauty salon**	Yes	114	95	1.14(0.77–1.68)	0.503
	No	103	98	Ref	
**Presence of hand-washing facility with water and soap**	Yes	154	49	7.18(4.64–11.12)	<0.001
	No	63	144	Ref	
**Presence of poster showing hand-washing behavior**	Yes	104	92	1.01(0.68–1.49)	0.958
	No	113	101	Ref	
**Presence of hand-washing facility that is convenient and user friendly in/near the barbershop/the beauty salon**	Yes	111	93	1.12(0.76–1.66)	0.549
	No	106	100	Ref	

Ref, reference category; COR, crude odds ratio; CI, confidence interval

From multivariable logistic regression analysis, female sex, educational level of college or above, positive attitude towards taking precautions against COVID-19, belief in the effectiveness of hand hygiene practices and presence of a hand-washing facility with soap and water showed significant association with practice of good hand hygiene among barbers and beauty salon worker (Table 7).

**Table 7:** Factors associated with good hand hygiene practice from multivariable logistic regression analysis among barbers and beauty salon workers in Dessie City and Kombolcha Town, Northeastern Ethiopia, January 5 to February 10, 2021.

Variables	Response	Hand hygiene practice		AOR (95%CI)	*P-*value
		Good	Poor		
		n	n		
**Sex**	Male	99	151	Ref	
	Female	118	42	2.17(1.29–3.65)	0.003
**Educational level**	Primary (up to grade 8)	87	89	Ref	
	Secondary (grades 9–12)	50	73	1.79(0.97–3.29)	0.061
	College or above	80	31	5.53(2.85–10.71)	<0.001
**Attitude towards taking precautions against COVID-19**	Positive	159	85	2.47(1.46–4.17)	0.001
	Negative	58	108	Ref	
**Belief in the effectiveness of hand hygiene**	Yes	182	85	3.78 (2.18–6.55)	<0.001
	No	35	108	Ref	
**Presence of hand-washing facility with soap and water**	Yes	154	49	5.55 (3.28–9.40)	<0.001
	No	63	144	Ref	

Ref, reference category; AOR, adjusted odds ratio; CI, confidence interval

We found that the odds of practicing good hand hygiene among female barbers and beauty salon workers were 2.17 times (AOR = 2.17, 95% CI: 1.29–3.65) higher than male barbers and beauty salon workers. The odds of developing good hand hygiene practice among barbers and beauty salon workers with an educational level of college or above were 5.53 times (AOR = 5.53, 95% CI: 2.85–10.71) higher than those with a lower level of education. On the other hand, the odds of practicing good hand hygiene among individuals with a positive attitude towards taking precautions against COVID-19 were 2.4 times (AOR = 2.4, 95% CI:1.46–4.17) higher than those with a negative attitude towards taking such precautions.

Similarly, the odds of practicing good hand hygiene among barbers and beauty salon workers who believed in the effectiveness of hand hygiene practices were 3.78 times (AOR = 3.78, 95% CI: 2.18–6.55) higher than those who did not believe in the effectiveness of hand hygiene. Furthermore, the odds of practicing good hand hygiene among barbers and beauty salon workers were 5.5 times (AOR = 5.55, 95% CI: 3.28–9.40) higher than those whose shops/salons had no hand-washing facility (Table 7).

## Discussion

Hand hygiene is usually used as a second factor for controlling the spread of disease if contact occurs [34]. But in the case of barbers and beauty salon workers for whom contact is mandatory, hand hygiene is the best option. Therefore, this study was conducted to assess hand hygiene practices and associated factors among barbers and beauty salon workers in Dessie City and Kombolcha Town. We found that 52.9% of the barbers and beauty salon workers had good hand hygiene practices. Our findings showed that good hand hygiene practice was significantly associated with sex, educational level, attitude towards taking precautions against COVID-19, belief in the effectiveness of hand hygiene practices and the presence of a hand-washing facility with soap and water.

Hand hygiene has been known to prevent respiratory infections. During SARS and H1N1 influenza outbreaks, hand hygiene with soap and water or alcohol-based hand sanitizer played a significant role in the reduction of the outbreaks [35–37]. Similarly, hand hygiene has been proven to prevent the transmission of COVID-19 [38–40]. Although there is evidence that hand hygiene can reduce respiratory diseases, in our study, only 52.9% of the barbers and beauty salon workers practised good hand hygiene. The result was lower than that found by a study in a similar area among taxi drivers, which was 66.4%. This difference may have been due to the difference in study period and type of question used [29]. The result was also lower than found in other studies from Ethiopia (82%), (76%) and (95.5%) [41–43], Nigeria (95.3%) and (69.9%) [44, 45], Malaysia (87.8%) [46], Poland (58.4%) [21], Japan (58.5%) [47], China (79.44%) [48], and United States (85.2%) [49]. The possible reason for the lower proportion of good hand hygiene in our study area might have been due to the fact that our study was conducted at the later stage of the outbreak when compliance with hand hygiene recommendations had gone down.

This finding was supported by a recent study where compliance with hand hygiene recommendations was lower in a later stage of the outbreak [19]. This reduction in compliance during the later stage of the outbreak might be due to adaptation to the disease and the presence of vaccines. However, this study reports a higher proportion of good hand hygiene practice than found by previous studies from Ethiopia (14.9%) and (43.0%) [50, 51], China (42.05%) [52], Indonesia (27.1%) [53], Vietnam (31%) [54], Turkey (42.4%) [55] and from a systematic review finding (40%) [56]. The reason for this discrepancy might be the difference in the socioeconomic status, different scoring systems and the type of questions used. The difference could also be due to the fact that this study was conducted at a time when the cost of alcohol-based hand sanitizer had gone down compared with the cost during the earlier stage of the outbreak.

In the present study, being female showed a significant association with good hand hygiene practices. Similar results were found by previous studies in Ghana [57], China [52], Switzerland [58], Turkey [55, 59], United States [49], Poland [60], Korea [61, 62] and the result from a review of studies [32, 33, 63, 64]. This could be due to a greater perceived susceptibility to disease amongst women compared to men. It could also be due to the presence of water and soap for hair washing purposes in female beauty salons being more common compared with male barbershop where hair washing services are less common.

Educational level showed a direct association with hand hygiene practices among barbers and beauty salon workers. This result was supported by recent studies in Kenya [65], China [52], Turkey [59], Vietnam [66], Switzerland [58] and a review of studies [32, 33]. The possible reason for the association of educational level with hand hygiene practices might have been that having a higher educational level influences the ability to seek and understand health information and actions to prevent COVID-19. However, an inverse relationship between educational level and hand hygiene practices was observed in studies conducted in Hong Kong [67, 68]. The reason for this might be due to the variation in socio-demographic characteristics of the population.

In this study, a positive attitude towards taking precautions against COVID-19 among barbers and beauty salon workers was positively associated with good hand hygiene practices. Barbers and beauty salon workers with a positive attitude towards taking precautions against COVID-19 were 2.4 times more likely to practice good hand hygiene compared with those with a negative attitude towards taking precautions against COVID-19. Consistent results were shown in recent studies in the United States [49, 69] and China [33]. The reason might be that those barbers and beauty salon workers with positive attitudes felt compelled to practice good hand hygiene since attitude is the driving force for practices.

Our study also found an association of hand hygiene practices with belief in the effectiveness of hand hygiene. In this study, barbers and beauty salon workers who believed in the effectiveness of hand hygiene in the prevention of COVID-19 were 3.7 times more likely to practice good hand hygiene than those who did not have this belief. This result is supported by studies in Korea [61, 62], England [70] and Hong Kong [71, 72]. The possible explanation might be the fact that belief is an influential determining factor of good practices.

Over 2 billion people in the world lacked a hand-washing facility with soap and water. In sub-Saharan Africa, more than 50% of the population are without a hand-washing facility [73]. Similarly, in our study only 49% of barbershops and beauty salons had a hand-washing facility with soap and water. This hinders practice of hand hygiene, and as a result, it promotes the spread of COVID-19. In this study, workers in barbershops and beauty salons with a hand-washing facility were 5.5 times more likely to practice good hand hygiene than those in shops and salons with no hand-washing facility. Similar findings have been reported by other studies [23, 74]. This could be due to the fact that the barbers and beauty salon workers are alarmed by the virus and motivated by the presence of a hand-washing facility.

## Limitations of the study

This study had several limitations. Although the presence or absence of a hand-washing facility with soap and water and alcohol-based hand sanitizer was determined by the data collectors’ observation, the proportion of workers following good hand hygiene practices was determined based on self-report that was not verified using direct observation, and therefore was subject to recall and social desirability biases [75]. In addition, since the study was conducted only in Dessie City and Kombolcha Town, the finding is not generalizable to all barbers and beauty salon workers at the national level. Moreover, due to limited access to studies on hand hygiene practices among barbers and beauty salon workers; the discussion was made on the basis of the findings with other target groups.

## Conclusion

This study showed that the proportion of barbers and beauty salon workers who practised good hand hygiene in Dessie City and Kombolcha Town was 52.9%. The predictors of good hand hygiene practices were sex, educational level, attitude towards taking precautions against COVID-19, belief in the effectiveness of hand hygiene practices and the presence of a hand-washing facility with soap and water. Therefore, it is recommended that training be provided for barbers and beauty salon workers to enhance their hand hygiene practices. In addition, government and non-government organizations should work together to provide alcohol-based hand sanitizer at a low cost to those shop/salon locations that are without access to a hand-washing facility with water and soap.

## Supporting information

S1 File English version of the questionnaire for hand hygiene practices during the COVID-19 pandemic and associated factors among barbers and beauty salon workers in Ethiopia. (DOCX)

S2 File Amharic version of the questionnaire hand hygiene practices during the COVID-19 pandemic and associated factors among barbers and beauty salon workers in Ethiopia. (DOCX)

S1 Data Data set for hand hygiene practices during the COVID-19 pandemic and associated factors among barbers and beauty salon workers in Ethiopia. (XLSX)

## Abbreviations

AOR: adjusted odds ratio
CI: confidence interval
COR: crude odds ratio
COVID-19: coronavirus infectious disease-19
