# Supplementary material for: Hand hygiene practices during the COVID-19 pandemic and associated factors among barbers and beauty salon workers in Ethiopia
Source: PLoS One. 2022 Jul 1;17(7):e0269225. doi: 10.1371/journal.pone.0269225 (PMC9249229; doi:10.1371/journal.pone.0269225)
Supplement: S1 File — (DOCX) [file pone.0269225.s001.docx]

**Questionnaire of the study**

**Part one (100) -: Socio-demographic and economic characteristics**

| Code | **Questions** | **Response** |
| --- | --- | --- |
| 101 | Sex | 1. Male B. Female |
| 102 | Age (years) | __________________ |
| 103 | Educational level | __________________ |
| 104 | Marital status | 1. Single C. Divorced 2. Married D. Widowed |
| 105 | Place of residence | A. Urban B. Rural |
| 106 | Monthly income | __________________ |
| 107 | Household size | __________________ |
| 108 | Number of workers in the barbershop / beauty salons | __________________ |
| 109 | Number of customers per day | __________________ |
| 110 | Work experience (years) | __________________ |
| 111 | Training about COVID-19 | 1. Yes B. No |
| 112 | Availability of COVID-19 IPC guideline | 1. Yes B. No |
| 113 | Has chronic illness | 1. Yes B. No |

**Part Two (200):-Questions developed to assess the knowledge of the respondents about COVID-19**

| Code | **Questions** | **Response** | |
| --- | --- | --- | --- |
|  |  | **Yes** | **No** |
| 201 | The main clinical symptoms of COVID-19 are fever, fatigue, dry cough, and myalgia |  |  |
| 202 | COVID-19 is transmitted through droplets when an infected person coughs, sneezes or speaks |  |  |
| 203 | COVID-19 is transmitted by touching a contaminated surface and then touching your eyes, nose or mouth |  |  |
| 204 | The disease could be transmitted from an asymptomatic person |  |  |
| 205 | The virus may be more dangerous in patients with chronic diseases |  |  |
| 206 | The virus may be more dangerous for the elderly |  |  |
| 207 | An effective vaccine against the virus is currently available |  |  |
| 208 | Washing hands frequently with soap and water for at least 20 seconds or using an alcohol-based hand sanitizer (60%) is important to prevent infection with COVD-19 |  |  |
| 209 | Wearing facemasks when moving out of home is important to prevent the infection with COVID-19 virus |  |  |
| 210 | To prevent the COVID-19 infection, individuals should avoid going to crowded places |  |  |
| 211 | Travelling to an infectious area or having contact with someone travelled to an area where the infection is present is a risk for developing an infection |  |  |
| 212 | Isolation and treatment of people who are infected with the COVID-19 virus are effective ways to reduce the spread of the virus |  |  |
| 213 | People who have contact with someone infected with the COVID-19 virus should be immediately isolated in a proper place |  |  |
| 214 | Children and young adults do not need to take measures to prevent the infection by the COVID-19 virus |  |  |

**Part Three (300):- Questions to assess the attitude of the respondents towards** **taking precautions against COVID-19**

| Code | **Questions** | **Response** |
| --- | --- | --- |
| 301 | Are you willing to do a voluntary test for COVID 19? | 1.Strongly Agree  2.Agree  3.Neutral  4.Disagree  5.Strongly Disagree |
| 302 | It is crucial to report a suspected case to health authorities? | 1.Strongly Agree  2.Agree  3.Neutral  4.Disagree  5.Strongly Disagree |
| 303 | It is important to use a facemask in a crowded place? | 1.Strongly Agree  2.Agree  3.Neutral  4.Disagree  5.Strongly Disagree |
| 304 | Is it important to wash hands after coming outsides? | 1.Strongly Agree  2.Agree  3.Neutral  4.Disagree  5.Strongly Disagree |
| 305 | Do you think COVID-19 is a preventable disease? | 1.Strongly Agree  2.Agree  3.Neutral  4.Disagree  5.Strongly Disagree |
| 306 | Do you think lack of knowledge about the mode of transmission and infection control measures contributes to increased risk of COVID-19**?** | 1.Strongly Agree  2.Agree  3.Neutral  4.Disagree  5.Strongly Disagree |
| 307 | Do you usually follow the updates about the spread of the virus in your country and worldwide? | 1.Strongly Agree  2.Agree  3.Neutral  4.Disagree  5.Strongly Disagree |
| 308 | If a lecture about the virus is organized near you, would you be willing to attend it? | 1.Strongly Agree  2.Agree  3.Neutral  4.Disagree  5.Strongly Disagree |
| 309 | If flyers or brochures that include information about the disease are distributed, would you be willing to read them and follow the instructions mentioned in them? | 1.Strongly Agree  2.Agree  3.Neutral  4.Disagree  5.Strongly Disagree |
| 310 | If protective measures and equipment are available at an affordable price, would you be willing to buy them? | 1.Strongly Agree  2.Agree  3.Neutral  4.Disagree  5.Strongly Disagree |
| 311 | Are you willing to do a voluntary test for COVID 19? | 1.Strongly Agree  2.Agree  3.Neutral  4.Disagree  5.Strongly Disagree |
| 312 | It is crucial to report a suspected case to health authorities? | 1.Strongly Agree  2.Agree  3.Neutral  4.Disagree  5.Strongly Disagree |
| 313 | It is important to use a facemask in a crowded place? | 1.Strongly Agree  2.Agree  3.Neutral  4.Disagree  5.Strongly Disagree |
| 314 | Is it important to wash hands after coming outsides? | 1.Strongly Agree  2.Agree  3.Neutral  4.Disagree  5.Strongly Disagree |
| 315 | Do you think COVID-19 is a preventable disease? | 1.Strongly Agree  2.Agree  3.Neutral  4.Disagree  5.Strongly Disagree |

**Part Four (400):-Questions to assess behavioral and environmental factors**

| Code | **Questions** | **Response** | |
| --- | --- | --- | --- |
|  |  | **Yes** | **No** |
| 401 | Do you believe in the effectiveness of hand hygiene in preventing COVID-19? |  |  |
| 402 | Do you believe that there are no curative treatments for COVID-19? |  |  |
| 403 | Do you perceive yourself as vulnerable to COVID-19? |  |  |
| 404 | Do you worry about COVID-19? |  |  |
| 405 | Do you perceive that the consequence of getting COVID-19 is serious? |  |  |
| 406 | Do you experience any respiratory infection symptoms? |  |  |
| 407 | Do you have any acquaintances that experienced any respiratory infection symptoms? |  |  |
| 408 | Do you know someone who had positive test results for COVID-19**?** |  |  |
| 409 | Do you know someone who was hospitalized for severe illness or died from COVID-19? |  |  |
| 410 | Do you have young children in the household? |  |  |
| 411 | Do your family members encourage you to wash your hands? |  |  |
| 412 | Do healthcare workers encourage you to wash your hands? |  |  |
| **Workers living environment-related factors** | | | |
| 413 | Presence of water source close to the barbershop/ beauty salon (observation) |  |  |
| 414 | Do you experience a shortage of water? |  |  |
| 415 | Presence of improved latrine in/near the barbershop/beauty salon (observation) |  |  |
| 416 | Presence of privately owned latrine in/near the barbershop/beauty salon (observation) |  |  |
| 417 | Presence of hand-washing facility with water and soap (observation) |  |  |
| 418 | Presence of poster showing hand-washing behaviour (observation) |  |  |
| 419 | Presence of hand-washing facility which is convenient and user friendly in/near the barbershop/beauty salon (observation) |  |  |

**Part Five (500): Questions to evaluate hand hygiene practices**

| Code | **Questions** | **Response** |
| --- | --- | --- |
| 501 | 1.Hand hygiene practice before putting on a facemask | A. Always B. Sometimes C. Never |
|  | 2. Hand hygiene practice after removing a facemask | A. Always B. Sometimes C. Never |
|  | 3. Hand hygiene practice after coughing, sneezing, or blowing nose | A. Always B. Sometimes C. Never |
|  | 4. Hand hygiene practice after coming in contact with frequently touched surfaces/objects | A. Always B. Sometimes C. Never |
|  | 5. Hand hygiene practice after coming in contact with coins/birr notes | A. Always B. Sometimes C. Never |
|  | 6. Hand hygiene practice after using a latrine | A. Always B. Sometimes C. Never |
|  | 7. Hand hygiene practice before eating | A. Always B. Sometimes C. Never |
|  | 8.Hand hygiene practice after using public transportation | A. Always B. Sometimes C. Never |
|  | 9. Hand hygiene practice after returning home | A. Always B. Sometimes C. Never |
|  | 10. Hand hygiene practice before providing service to customers | A. Always B. Sometimes C. Never |
|  | 11. Hand hygiene practice after providing service to customers | A. Always B. Sometimes C. Never |
| 502 | Presence of alcohol-based hand sanitizer inside the barbershop/ beauty salon (observation) | 1. No B. Yes |
| 503 | Duration of hand hygiene procedure (seconds) | ___________________ |
| 504 | Frequency of washing hands (per day) | ___________________ |
| 505 | Do the worker demonstrates practical hand-washing procedure perfectly (observation) | 1. No B. Yes |

**Thank you!**
