# Supplementary material for: Hand hygiene practices during the COVID-19 pandemic and associated factors among barbers and beauty salon workers in Ethiopia
Source: PLoS One. 2022 Jul 1;17(7):e0269225. doi: 10.1371/journal.pone.0269225 (PMC9249229; doi:10.1371/journal.pone.0269225)
Supplement: S2 File — (DOCX) [file pone.0269225.s002.docx]

**ክፍል አንድ(100)፡ ማህበራዊና ኢኮኖሚያዊ ሁኔታዎች**

| **ኮድ** | **መጠይቅ** | **መልሶች** | |
| --- | --- | --- | --- |
| 101 | የመረጃ ሰጪው ፆታ | 1**.** ወንድ **2.** ሴት | |
| 102 | የመረጃ ሰጪው ዕድሜ**(**በዓመት) | __________________ | |
| 103 | የትምህርት ሁኔታ | __________________ | |
| 104 | የጋብቻ ሁኔታ | 1. ያላገባ 3. ሚስቱ በሞት የተለየችበት  2. ያገባ 4. የፈታ | |
| 105 | የመኖርያ አድራሻ | 1. ከተማ 2. ገጠር | |
| 106 | የቤተሰቡ ወርሃዊ የገቢ መጠን | __________________ | |
| 107 | የቤተሰብ ብዛት | __________________ | |
| 108 | የሰራተኞች ብዛት | __________________ | |
| 109 | ህለታዊ አማካኝ የደንበኞች ብዛት | __________________ | |
| 110 | የመረጃ ሰጪው የስራ ልምድ**(**በዓመት) | __________________ | |
| 111 | ኮሮና ቫይረስን በተመለከተ ስልጠና ወስደሃል/ሻል? | | 0. የለም 1. አዎ |
| 112 | የኮሮና ቫይረስ መከላከላከያ መንገዶችን የሚያሳይ ስህላዊ መግለጫ አለ? (በምልከታ የሚመለስ) | | 0. የለም 1. አዎ |
| 113 | ስር የሰደዱ በሽታዎች እንደ ደም ግፊት፤ ስኳር በሽታ አሉብህ/ሽ? | | 0. የለም 1. አዎ |

**ክፍል ሁለት (200):- የጥናቱ ተሳታፊዎችን የኮሮና ቫይረስ የዕዉቀት ደረጃ ለመለካት የተዘጋጀ መጠይቅ**

| **ኮድ** | **መጠይቅ** | **መልሶች** |
| --- | --- | --- |
| 201 | የኮሮና ቫይረስ ዋነኛ ምልክቶች ትኩሳት፤ ደረቅ ሳል ፤ድካምና የጡንቻ ህመም ናቸዉ፡፡ | 0. የለም 1. አዎ |
| 202 | የኮሮና ቫይረስ የሚሰራጨዉ በበሽታዉ የተያዘ ሰዉ በሚስልበት፤ በሚያስነጥስበት ወይንም በሚወራበት ጊዜ በሚወጡ ጠብታዎች ነዉ፡፡ | 0. የለም 1. አዎ |
| 203 | የኮሮና ቫይረስ የሚሰራጨዉ በቫይረሱ የተበከለ ቦታን ነክቶ ባልታጠበ እጅ አይንን፤ አፍንጫንና አፍህን በመንካት ነዉ፡፡ | 0. የለም 1. አዎ |
| 204 | የኮሮና ቫይረስ የበሽታው ምልክት በማይታይባቸው ሰወች ሊተላለፍ ይችላል፡፡ ፡፡ | 0. የለም 1. አዎ |
| 205 | የኮሮና ቫይረስ ተጉዳኝ በሽታዎች ያሉባቸዉ ስዎች ላይ በጣም አደገኛ ነዉ፡፡ | 0. የለም 1. አዎ |
| 206 | የኮሮና ቫይረስ በእድሜ የገፋ ስዎች ላይ በጣም አደገኛ ነዉ፡፡ | 0. የለም 1. አዎ |
| 207 | የኮሮና ቫይረስ ክትባት አለው | 0. የለም 1. አዎ |
| 208 | እጅን በሳሙናና በዉሃ ለ20 ሰከንድ መታጠብ ወይንም የእጅ ማፅጃ ሳኒታይዘር መጠቀም ቫይረሱን ለመከላከል ጠቃሚ ነዉ፡፡ | 0. የለም 1. አዎ |
| 209 | ከቤት ውጭ በምንቀሳቀስበት ጊዜ የአፍና አፍንጫ መሸፈኛ ጭንብል ማድረግ ቫይረሱን ለመከላከል ሁነኛ መንገድ ነዉ፡፡ | 0. የለም 1. አዎ |
| 210 | የቫይረሱን ስርጭት ለመግታት የተጭናነቁና ሕዝብ የሚበዛባቸዉ ቦታዎች ከመሄድ መቆጠብ አለብን፡፡ | 0. የለም 1. አዎ |
| 211 | ቫይረሱ የሚበዛባቸው ቦታወች በመሄድ ወይንም ቫይረሱ የሚበዛባቸው ቦታወች ከሄደ ሰዉ ጋር መገናኘት ለበሽታዉ እንድንጋለጥ ያደርገናል፡፡ | 0. የለም 1. አዎ |
| 212 | በቫይረሱ የተያዙ ሰዎች ላይ የሚደረገዉ መለየትና ህክምና የበሽታዉን ስርጭት ለመግታት ይረዳል ፡፡ | 0. የለም 1. አዎ |
| 213 | በቫይረሱ መያዙ የተረጋገጠ ሰዉ በአፋጣኝ ራሱን መለየት አለበት ፡፡ | 0. የለም 1. አዎ |
| 214 | ልጆችና አዋቂዎች በቫይረሱ ላለመያዝ ጥንቃቄ ማድረግ አይጠበቅባቸዉም፡፡ | 0. የለም 1. አዎ |

**ክፍል 3(300) ፡- የጥናቱ ተሳታፊዎችን የኮሮና ቫይረስ የዕይታ ደረጃን ለመለካት የተዘጋጀ መጠይቅ**

| **ኮድ** | **መጠይቅ** | **መልሶች** |
| --- | --- | --- |
| 301 | ቫይረሱን ለመለየት የሚደረግ ላቦራቶሪ ምርመራ ካለ ለማድረግ ፍቃደኛ ነህ? | 1. በጣም እስማማለሁ  2. እስማማለሁ  3. ገለልተኛ  4. አልስማማም  5. በጣም አልስማማም |
| 302 | በበሽታዉ የተጠረጠረን ሰዉ ለጤና ባለሞያዎች ማሳወቅ አስፈላጊ ነው? | 1. በጣም እስማማለሁ  2. እስማማለሁ  3. ገለልተኛ  4. አልስማማም  5. በጣም አልስማማም |
| 303 | በተጨናነቀ ቦታ ላይ የአፍና አፍንጫ መሸፈኛ ጭንብል ማድረግ አስፈላጊ ነው? | 1. በጣም እስማማለሁ  2. እስማማለሁ  3. ገለልተኛ  4. አልስማማም  5. በጣም አልስማማም |
| 304 | ከውጭ ወደ ቤት ከተመለስን በኃላ እጅ መታጠብ አስፈላጊ ነው? | 1. በጣም እስማማለሁ  2. እስማማለሁ  3. ገለልተኛ  4. አልስማማም  5**.** በጣም አልስማማም |
| 305 | የኮሮና ቫይረስ መከላከል ምንችለው በሽታ ነው ብለህ ታስባለህ? | 1. በጣም እስማማለሁ  2. እስማማለሁ  3. ገለልተኛ  4. አልስማማም  5**.** በጣም አልስማማም |
| 306 | ስለኮሮና ቫይረስ መተላለፊያና መከላከያ መንገዶች በቂ እውቀት ማጣት በቫይረሱ የመያዝ ዕድልን ይጨምራል ብለህ ታስባለህ? | 1. በጣም እስማማለሁ  2. እስማማለሁ  3. ገለልተኛ  4. አልስማማም  5**.** በጣም አልስማማም |
| 307 | ስለቫይረሱ በየጊዜዉ የሚሰጡ ሀገራዊና ዓለማቀፋዊ መረጃዎችን ትከታተላለህ? | 1**.** በጣም እስማማለሁ 2.እስማማለሁ  3. ገለልተኛ  4**.** አልስማማም  5**.** በጣም አልስማማም |
| 308 | ቫይረሱን በተመለከተ የሚዘጋጁ ትምህርታዊ ዝግጅቶችን ትከታተላለህ? | 1**.** በጣም እስማማለሁ  2. እስማማለሁ  3. ገለልተኛ  4**.** አልስማማም  5**.** በጣም አልስማማም |
| 309 | ስለቫይረሱ መረጃ የያዙ በራሪ ወረቀቶችን ስታገኝ ታነባለህ መመርያዎቹንም ትከተላለህ? | 1. በጣም እስማማለሁ  2. እስማማለሁ  3. ገለልተኛ  4. አልስማማም  5**.** በጣም አልስማማም |
| 310 | ቫይረሱን ለመቆጣጠር የሚሆኑ መሳርያዎች በተመጣጣኝ ዋጋ ከተገኘ ትገዛለህ? | 1. በጣም እስማማለሁ  2. እስማማለሁ  3. ገለልተኛ  4. አልስማማም  5. በጣም አልስማማም |

**ክፍል 4(400) ፡- የእጅህን ንጽህና** **አጠባበቅ ባህሪያት ጋር የተያያዙ ሁኔታዎች**

| **ኮድ** | **መጠይቅ** | **መልሶች** |
| --- | --- | --- |
| 401 | እጅን መታጠብ የኮሮና ቫይረስ ስርጭትንና መተላለፍን ይከላከላል ብለህ ታምናለህ? | 0. የለም 1. አዎ |
| 402 | የኮሮና ቫይረስ መድሀኒት እንደሌለው ታምናለህ? | 0. የለም 1. አዎ |
| 403 | ለኮሮና ቫይረስ ተጋላጭ ነኝ ብለህ ታስባልህ? | 0. የለም 1. አዎ |
| 404 | ስለኮሮና ቫይረስ ትጨነቃለህ? | 0. የለም 1. አዎ |
| 405 | በኮሮና ቫይረስ መያዝ የሚያስከትለው ችግር ከባድ ነዉ ብለሀ ታስባልህ? | 0. የለም 1. አዎ |
| 406 | በቅርብ ጊዜ ዉስጥ የመተንፈሻ አካላት በሽታ ምልክቶች ታይተውብሀል? | 0. የለም 1. አዎ |
| 407 | የመተንፈሻ አካላት በሽታ ምልክቶች የሚታይበት የቅርብ ዘመድ አለህ? | 0. የለም 1. አዎ |
| 408 | በኮሮና ቫይረስ የተዘ ሰው ታውቃለህ? | 0. የለም 1. አዎ |
| 409 | በኮሮና ቫይረስ ምክንያት ለጠና ህመም ሆስፒታል የገባ ወይንም የሞተ ሰው ታውቃለህ? | 0. የለም 1. አዎ |
| 410 | በቤትህ ዉስጥ ህፃናት አሉ? | 0. የለም 1. አዎ |
| 411 | ቤተሰቦችህ እጅህን እንድትታጠብ ያበረታቱሃል? | 0. የለም 1. አዎ |
| 412 | የጤና ባለሞያዎች እጅህን እንድትታጠብ ያበረታቱሃል? | 0. የለም 1. አዎ |
| **የዉበት ሳሎኑ ሰራተኞች የመኖሪያ አከባቢን በተመለከተ** | | |
| 413 | በዉበት ሳሎኑ አጠገብ የቧንቧ ውሀ አለ?(በምልከታ የሚመለስ) | 0. የለም 1. አዎ |
| 414 | የዉሀ እጥረት/መቆራረጥ አለ? | 0. የለም 1. አዎ |
| 415 | ደረጃዉን የጠበቀ/የተሻሻለ መፀዳጃ ቤት አለ?(በምልከታ የሚመለስ) | 0. የለም 1. አዎ |
| 416 | መፀዳጃ ቤቱ የግል ነው? | 0. የለም 1. አዎ |
| 417 | መፀዳጃ ቤት አጠገብ ውሃ ሳሙና ያለዉ የእጅ መታጠብያ አለ?(በምልከታየሚመለስ) | 0. የለም 1. አዎ |
| 418 | የእጅህ መታጠብን የሚያሳይ ስህላዊ መግለጫ አለ?(በምልከታ የሚመለስ) | 0. የለም 1. አዎ |
| 419 | ምቹ የሆነ የእጅህ መታጠብያ አለ? (መስታወት፣ሳሙና ማስቀመጫ ያለው) (በምልከታ የሚመለስ) | 0. የለም 1. አዎ |

**ክፍል 5(500) ፡- የእጅህን ንጽህና** **አጠባበቅ ጋር የተያያዙ ጥያቄዎች**

| **ኮድ** | **መጠይቅ** | **መልሶች** |
| --- | --- | --- |
| 501 | የእጅህን ንጽህና ምን ምን ተግባራትን ካከናወንክ በኃላ/በፊት ትጠብቃለህ? | |
|  | 1. ጭምብልን ከማድረግ በፊት | ሀ) ዘወትር ለ) አልፎ አልፎ ሐ) ጭራሽ |
|  | 2. ጭምብልን ካወለቅን በኃላ | ሀ) ዘወትር ለ) አልፎ አልፎ ሐ) ጭራሽ |
|  | 3. ከማሳል ከማስነጠስ ከተናፈጥን በኃላ | ሀ) ዘወትር ለ) አልፎ አልፎ ሐ) ጭራሽ |
|  | 4. በተደጋጋሚ የሚነኩ ህቃወችን/ ቦታወችን ከነካን በኃላ | ሀ) ዘወትር ለ) አልፎ አልፎ ሐ) ጭራሽ |
|  | 5. ሳንቲም/ብር ኖት ከነካን በኃላ | ሀ) ዘወትር ለ) አልፎ አልፎ ሐ) ጭራሽ |
|  | 6. መፀዳጃ ቤት ከተጠቀምን በኃላ | ሀ) ዘወትር ለ) አልፎ አልፎ ሐ) ጭራሽ |
|  | 7. ምግብ ከመመገባችን በፊት | ሀ) ዘወትር ለ) አልፎ አልፎ ሐ) ጭራሽ |
|  | 8. የህዝብ/የጋራ ትራነስፖርት ከተጠቀምን በኃላ | ሀ) ዘወትር ለ) አልፎ አልፎ ሐ) ጭራሽ |
|  | 9. ከዉጭ ወደ ቤት ከተመልስን በኃላ | ሀ) ዘወትር ለ) አልፎ አልፎ ሐ) ጭራሽ |
|  | 10. ለደንበኞች አገልግሎት ከመሰጠት በፊት | ሀ) ዘወትር ለ) አልፎ አልፎ ሐ) ጭራሽ |
|  | 11. ለደንበኞች አገልግሎት ከሰጠን በኃላ | ሀ) ዘወትር ለ) አልፎ አልፎ ሐ) ጭራሽ |
| 502 | የዉበት ሳሎኑ ውስጥ የእጅ ማፅጃ ሳኒታይዘር አለ?(በምልከታ የሚመለስ)? | 0. የለም 1. አዎ |
| 503 | እጅህን ለምን ያክል ጊዜ ትታጠባለህ?(በሰከንድ) | ___________________ |
| 504 | እጅህን በቀን ስንት ጊዜ ትታጠባለህ? | ___________________ |
| 505 | እጅ የመታጠቢያ ትክክለኛ ሂደት ማሳየት ይችላል? (በምልከታ የሚመለስ)? | 0. የለም 1. አዎ |

**አመሰግናለሁ!!!**
